# Changes in growth, physiology, and photosynthetic capacity of spinach (*Spinacia oleracea* L.) under different nitrate levels

**DOI:** 10.1371/journal.pone.0283787

**Published:** 2023-03-31

**Authors:** Kangning Han, Jing Zhang, Cheng Wang, Yan Yang, Youlin Chang, Yanqiang Gao, Yang Liu, Jianming Xie

**Affiliations:** College of Horticulture, Gansu Agricultural University, Lanzhou, Gansu, China; University of Agriculture Faisalabad-Pakistan, PAKISTAN

## Abstract

Nitrate content is an essential indicator of the quality of vegetables but can cause stress at high levels. This study aimed to elucidate the regulatory mechanisms of nitrate stress tolerance in spinach (*Spinacia oleracea* L.). We studied the effects of exogenous application of 15 (control), 50, 100, 150, 200, and 250 mM NO_3_^-^ on spinach growth, physiology, and photosynthesis. The results showed that all the nitrate treatments inhibited the growth of the aerial parts of spinach compared to the control. In contrast, low nitrate levels (50 and 100 mM) promoted spinach root formation, but this effect was inhibited at high levels (150, 200, and 250 mM). Treatment with 150 mM NO_3_^-^ significantly decreased the root growth vigor. Low nitrate levels increased the chlorophyll content in spinach leaves, whereas high levels had the opposite effect. High nitrate levels also weakened the net photosynthetic rate (Pn), the actual photochemical efficiency of PSII Y(II), and increased non-photochemical quenching (NPQ), reducing photosynthetic performance. Nitrate stress increased the activity of nitrate reductase (NR) and promoted the accumulation of nitrate in spinach leaves, exceeding the health-tolerance limit for nitrate in vegetables, highlighting the necessity of mitigating nitrate stress to ensure food safety. Starting with the 150 mM NO_3_^-^ treatment, the proline and malondialdehyde content in spinach leaves and roots increased significantly as the nitrate levels increased. Treatment with 150 mM NO_3_^-^ significantly increased soluble protein and flavonoid contents, while the activities of superoxide dismutase (SOD), peroxidase (POD), catalase (CAT), and ascorbate peroxidase (APX) were significantly reduced in leaves. However, spinach could resist nitrate stress by regulating the synthesis of osmoregulatory substances such as proline, thus showing some nitrate tolerance. These results provide insights into the physiological regulatory mechanisms of nitrate stress tolerance and its mitigation in spinach, an essential vegetable crop.

## Introduction

Spinach (*Spinacia oleracea* L.) is a popular and nutritious vegetable from the Chenopodiaceae family [1]. It is a rich source of vitamin C, carotene, protein, flavonoids, lutein, folic acid, minerals, and other nutrients [1, 2]. Spinach is a green leafy vegetable commonly grown and consumed in China. It is considered one of the healthiest vegetables in the human diet and is cold-resistant and adaptable [3]. Spinach is also a good source of chlorophyll and is helpful for digestion [4, 5]. However, they can quickly accumulate nitrate, which affects human health [6].

China is one of the major saline countries in the world, with a large saline area that increase annually, which can seriously affect the rate of change and quality of saline soils. Improper irrigation and excessive use of chemical fertilizers can cause soil degradation and secondary salinization of good farmlands [7]. In recent years, with the continuous increase in vegetable cultivation facilities, farmers tend to use a large amount of nitrogen fertilizer to increase the yield of vegetables. This is due to the nature of the environment of most protected greenhouses, which is characterized by high surface temperature, evaporation, and no rain, coupled with continuous cultivation and other inappropriate methods, leading to secondary salinization of the soil [8]. The excessive use of these nitrogen fertilizers leads to the accumulation of nitrate in plants and soils, which weakens plant resistance, inhibits growth and development, affects the safety and quality of agricultural products, and severely limits agricultural production [9, 10]. Nitrate can be converted to nitrite in the digestive tract but the consumption of vegetables with high nitrate content is potentially hazardous to health [11]. If saline soil can be rationally utilized, it can promote vegetable production and the problem of insufficient vegetable land can be alleviated. Therefore, improving the salt tolerance of vegetable crops is an effective way to make rational use of saline soil, which is of great significance for improving the quality of vegetable products and providing potential nutrients for humans.

Salt stress is a critical environmental factor because it causes an imbalance in plant nutrition, water deficit, oxidative stress, and disruption of cellular ion homeostasis [12, 13]. Spinach is known to be tolerant to mild salinity [14, 15] and accumulates salt in the leaf cells, but not in the apoplast [16]. Salt stress can affect the nutritional quality and promote physiological changes in spinach [17, 18]. Most early studies on salt stress have focused on NaCl [19, 20]. However, there have been few investigations on nitrate stress in spinach. It has been reported that excessive accumulation of nitrate inhibits the growth and development of vegetable crops in protected farmlands in China [21], However, little is known about the effect of nitrate on physiological mechanisms. Thus, in this study, changes in growth and physiological characteristics of spinach under different levels of nitrate stress were investigated to explore the physiological role of plants against nitrate stress in order to further understand the importance of reducing nitrate stress and provide a theoretical basis for it.

## Materials & methods

### Plant material

The spinach variety Fire Phoenix 119 was obtained from Tuochetou International Co., Ltd. in Hebei, China. Experimental trials were conducted at the College of Horticulture, Gansu Agricultural University, Lanzhou (36°03′N, 103°40′E), China. Spinach seeds with whole grains and uniform size were selected, rubbed, dispersed to remove spines, and then cold treated at 4 °C for 24 h to break dormancy. The seeds were then disinfected in 5% NaClO solution for 30 min, washed with deionized water, immersed in clean water, and shaken on a shaker for 12 h. The seeds were wrapped in wet towels and placed in an artificial climate box under dark conditions at 18 °C to accelerate uniform germination. Germinated seeds were planted in a perforated tray containing vermiculite and perlite in a ratio of 3:1 as seedling substrates. The seedlings were placed in an artificial climate box with a relative humidity of 70–80% and temperature of 20 °C and 16 °C under 12 h light (20000 Lx) and 12 h dark cycle, respectively. Seeds were watered with 1/8 Hoagland nutrient solution during substrate breaking to transplant. Then, spinach seedlings with four leaves and one heart with the same growth tendency were selected and transplanted into hydroponic boxes containing the Hoagland nutrient solution. The nutrient solution was changed every 5 days.

### Experimental design

Twenty days after transplantation, spinach plants of uniform growth and size were selected and treated with different nitrate concentrations until sampling. The nitrate stress test was conducted in a completely randomized design with a split-plot arrangement of three replicates. The following six treatments were used: Normal Hoagland nutrient solution (control), 50 mM NO_3_^-^ (T1), 100 mM NO_3_^-^ (T2), 150 mM NO_3_^-^ (T3), 200 mM NO_3_^-^ (T4), and 250 mM NO_3_^-^ (T5). The NO_3_^-^ concentration in the normal nutrient solution was 15 mM, the excess NO_3_^-^ in the stress treatment was provided by Ca (NO_3_) _2_·4H_2_O and KNO_3_, half each, and the pH was maintained at approximately 6.0. Based on the normal nitrate ion concentration, the required amount of nitrate ions was added to reach the treatment concentration. Plants were maintained using conventional methods, and all treatments were consistent. After one week of treatment, samples were collected, and the corresponding indices were determined.

### Growth parameters and biomass

After one week of treatment, the leaf length and width of each spinach plant were measured with a ruler, and each treatment was repeated five times. Spinach leaves and roots were scanned using an EPSON Expression 1100XL scanner (WinRHIZO ProLA2400, Canada), and photos were analyzed for leaf area, root length, root surface area, root volume, number of root tips, and branch number using Win RHIZO 5.0. Nine spinach plants were randomly harvested from each treatment group and divided into underground and aboveground parts. The fresh weight was first weighed separately, then oven-dried at 105 °C for 30 min, and then dried at 80 °C to obtain a constant dry weight, and the relevant indices were calculated.

### Root activity

The triphenyltetrazolium chloride (TTC) procedure was used to estimate root activity [22]. Then, 0.5 g of root tip sample was weighed into the test tube, added 5 ml each of phosphate buffer pH 7.0 and 0.4% TTC solution, held for 1 h at 37 °C, then added 2 ml of 1 M sulfuric acid was added to terminate the reaction, then removed the root system and filtered the water, put it into a mortar with 3 ml of ethyl acetate and ground it thoroughly, filtered it into a graduated test tube, washed the residue twice, fixed the volume to 10 ml. Meanwhile, the experiment required a set of blank tests; 2 mL of 1 M sulfuric acid was added first, followed by plant roots, and other operational steps remained unchanged. The blank test was first used as a standard for zeroing, and then, the absorbance value of the solution to be measured was read at 485 nm. Based on the standard curve, the TTC reduction was calculated by substituting it into the linear regression equation. The root reduction intensity was calculated according to the TTC reduction intensity equation. This was used to indicate root vigor strength.

### Calculations of photosynthetic pigment concentrations

Freshly shredded and mixed leaves were weighed to the nearest 0.1 g and placed at the bottom of a sealed 20 ml test tube. Then, 10 ml of 80% acetone was added, and the test tube was kept in the dark for 48 h with 12 h of shaking. When the leaves turned completely white, the optical density values of the extracted solutions were measured at 663 nm and 645 nm using a UV-1780 spectrometer (Shimadzu, Japan) and zeroed with acetone (80%). The contents of chlorophyll a, chlorophyll b, total chlorophyll, and carotenoids were calculated using the following equations:

$$\text{Chl}\;\text{a}\;\left(\text{mg}\cdot {\text{g}}^{-1}\text{FW}\right)=(12.71\times {\text{OD}}_{663}-2.59\times {\text{OD}}_{645})\times \left(\text{V}/1000\text{W}\right)$$

$$\text{Chl}\;\text{b}\;\left(\text{mg}\cdot {\text{g}}^{-1}\text{FW}\right)=(22.88\times {\text{OD}}_{645}-4.67\times {\text{OD}}_{663})\times \left(\text{V}/1000\text{W}\right)$$

$$\text{Total}\;\text{Chl}\;\left(\text{mg}\cdot {\text{g}}^{-1}\text{FW}\right)=\text{Chl}\;\text{a}+\text{Chl}\;\text{b}=(20.29\times {\text{OD}}_{645}+8.04\times {\text{OD}}_{663})\times \left(\text{V}/1000\text{W}\right)$$

V: Total volume of sample extraction solution (ml); W: Sample mass (g).

### Calculations of photosynthetic gas exchange parameters

The net photosynthetic rate (Pn), stomatal conductance (Gs), intercellular CO_2_ concentration (Ci), and transpiration rate (Tr) were measured using a CIRAS-2 portable photosynthesis sizer (PPSYSTEM, UK) from 9:00 am–11:00 am.

### Calculations of chlorophyll fluorescence parameters

After 7 days of nitrate treatment, three plants from each treatment were randomly selected from three replicates. After 30 min of dark acclimation, leaves of the same size and leaf position were selected to measure the chlorophyll fluorescence parameters of spinach leaves (ETR: electron transport rate; Fv/Fm: maximum photochemical quantum yield of PSII; NPQ: non-photochemical quenching; qP: photochemical quenching coefficient; Y(II): quantum efficiency of PSII photochemistry) using an Imaging-PAM chlorophyll fluorometer (Walz Effeltrich Germany) [23]. The detection parameters were set to 0.1 μmol m^-2^ s^-1^ for light detection, 111 μmol m^-2^ s^-1^ for photochemical light, 2700 μmol m^-2^ s^-1^ for saturation pulse light, 0.8 s for pulse light saturation time, and 20 s for time interval.

### Calculations of nitrate content and nitrate reductase (NR) activity

Nitrate content was determined using a colorimetric method [24]. NR activity was determined using an NR activity assay kit. (Kemin Biotechnology Co., Suzhou, China).

### Calculations of soluble protein, proline, malondialdehyde and flavonoids

The soluble protein content was determined using Coomassie’s brilliant blue G-250 method [25]. The proline content was determined using the acid ninhydrin method [26]. Malondialdehyde content was determined using the thiobarbituric acid method [27]. The flavonoid content was determined using the colorimetric method [28].

### Calculation of antioxidant enzyme activity

Superoxide dismutase (SOD) levels were determined using the method of Giannopolitis & Ries [29]. Peroxidase (POD) was determined using the method of Chance & Maehly [30]. Catalase (CAT) was determined using the method described by Aebi [31]. Ascorbate peroxidase (APX) was determined as described by Nakano & Asada [32].

### Statistical analysis

Data were analyzed by one-way analysis of variance (ANOVA) using the SPSS package program (version 20.0; SPSS Institute Ltd, USA). The experiment was repeated 3–5 times. Duncan’s test was used for significance (*p* < 0.05). Origin 2022 was used to draw graphs with standard error bars, and the values of the last graph are the average values of 3 or 5 repetitions.

## Results

### Plant morphology

**Fig 1:**
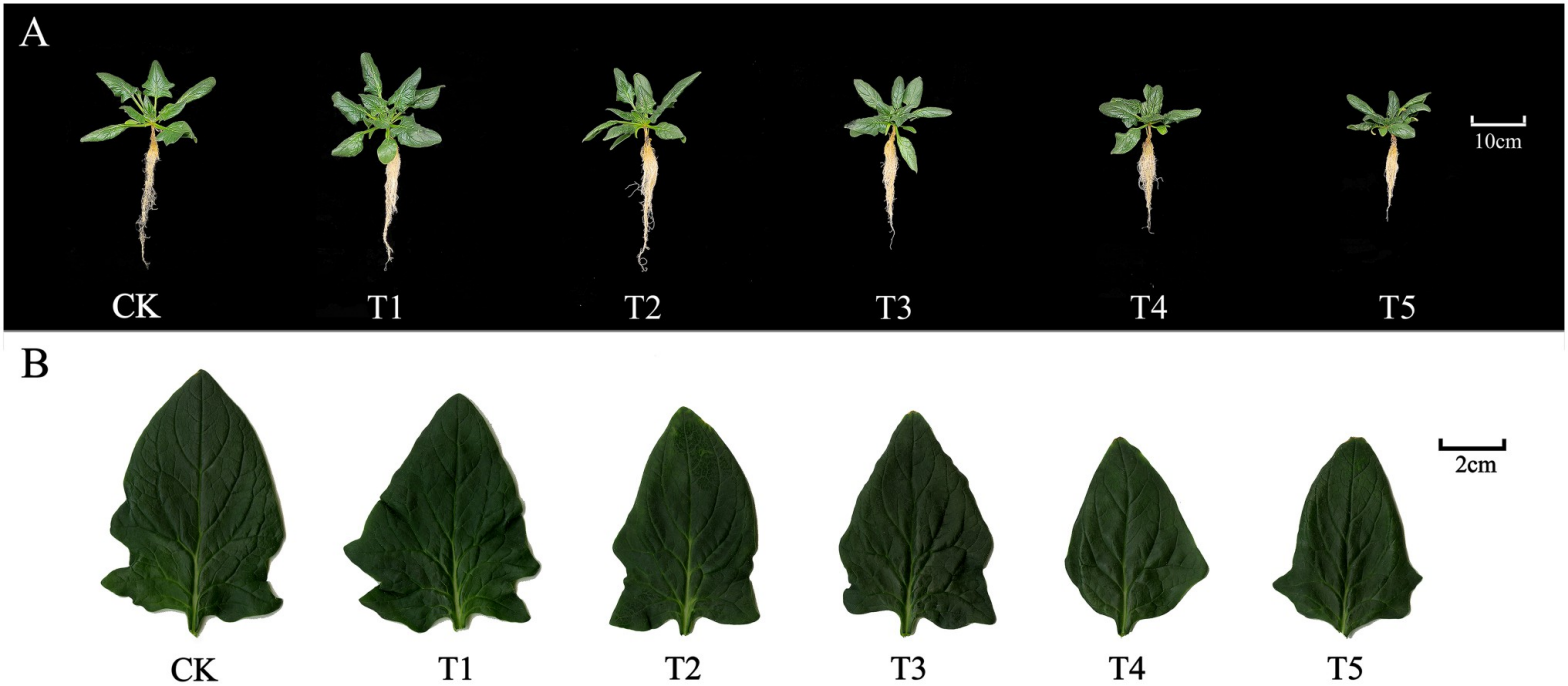
Effect of different nitrate levels on spinach.

Different nitrate treatments cause a distinctive shoot morphology. Hence, we decided to investigate the effect of different nitrate treatments (15 (control), 50, 100, 150, 200, and 250 mM NO_3_^-^) on the shoots and roots by photographing the whole plant body (Fig 1). The data showed that the application of lower doses (50 and 100 mM) had a positive effect on the spinach phenotype. In contrast, higher doses (150 to 250 mM) resulted in plant dwarfism and reduced leaf area. Hence, our data strongly suggest that nitrate exerts a dose-dependent effect on spinach morphology.

### Leaf growth parameters

Since the leaf is the major vegetative part of spinach, we focused on phenotyping various leaf-related parameters. Different nitrate concentrations significantly decreased the leaf length, width, and area of spinach (S1 Table). The leaf length of plants treated with T1, T2, T3, T4, and T5 decreased significantly compared to that of the control group. The leaf widths of T3, T4, and T5 decreased significantly by 8.30%, 9.81%, and 28.30%, respectively, compared to the control group. The leaf areas of the T4 and T5 treatments decreased significantly by 24.81% and 34.07%, respectively, compared to the control group.

### Biomass

The different nitrate treatments inhibited spinach biomass accumulation (S2 Table). Compared with the control group, treatments T2, T3, T4, and T5 significantly reduced the fresh weight of aboveground plant parts by 25.84%, 36.80%, 36.56%, and 37.60%, respectively. In contrast, treatment T5 significantly reduced the fresh weight of belowground parts and the dry weight of aboveground parts by 19.73% and 19.84%, respectively. There were no significant differences between the different nitrate treatments regarding the dry weight of the belowground parts. The relative water content in treatments T2, T3, T4, and T5 decreased significantly by 1.97%, 2.73%, 3.17%, and 3.19%, respectively, compared with the control group.

### Root system morphology

**Fig 2:**
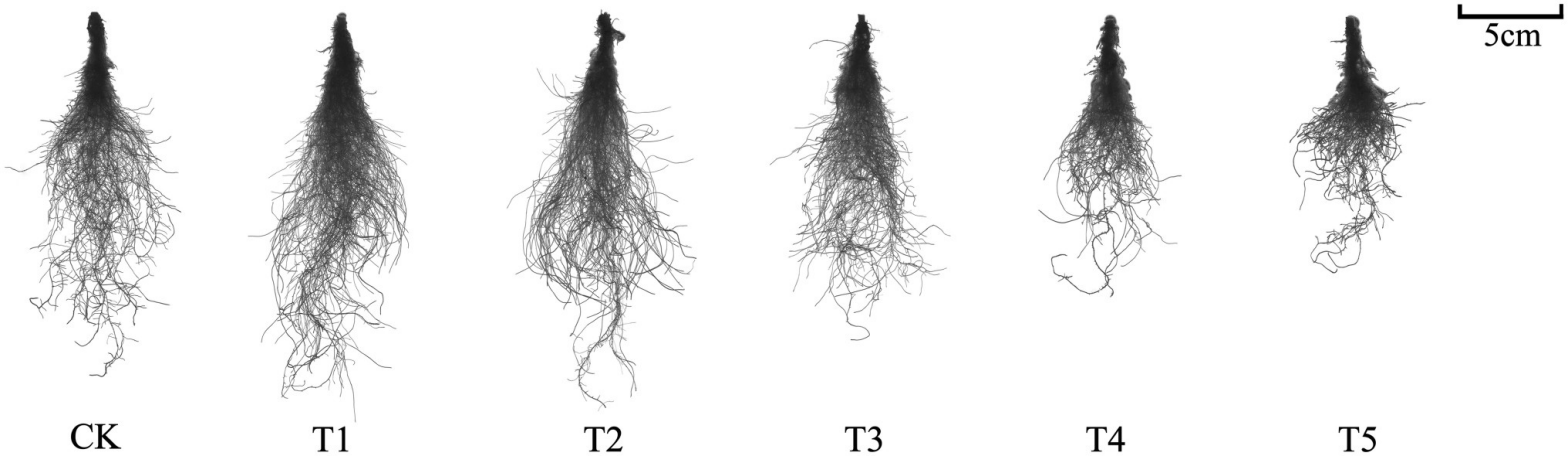
Effect of different nitrate levels on the morphology of spinach roots.

Spinach roots from the different treatments were washed and scanned using a root scanner, and the root system architecture was analyzed. Treatments T1 and T2 promoted spinach root growth. In contrast, treatments T3, T4, and T5 significantly inhibited root growth compared to the control group (Fig 2). The total root length, volume, surface area, and the number of branches of spinach roots first increased and then decreased with increasing nitrate concentration (S3 Table). The inflection point of each parameter occurred in the T3 treatment. Compared to the control group, total root length and total root volume significantly decreased by 18.45% and 9.80%, respectively, in the T3 treatment, while total root surface area, the number of root tips, and the number of branches decreased by 11.68%, 9.65%, and 4.60%, respectively.

### Root vigor

**Fig 3:**
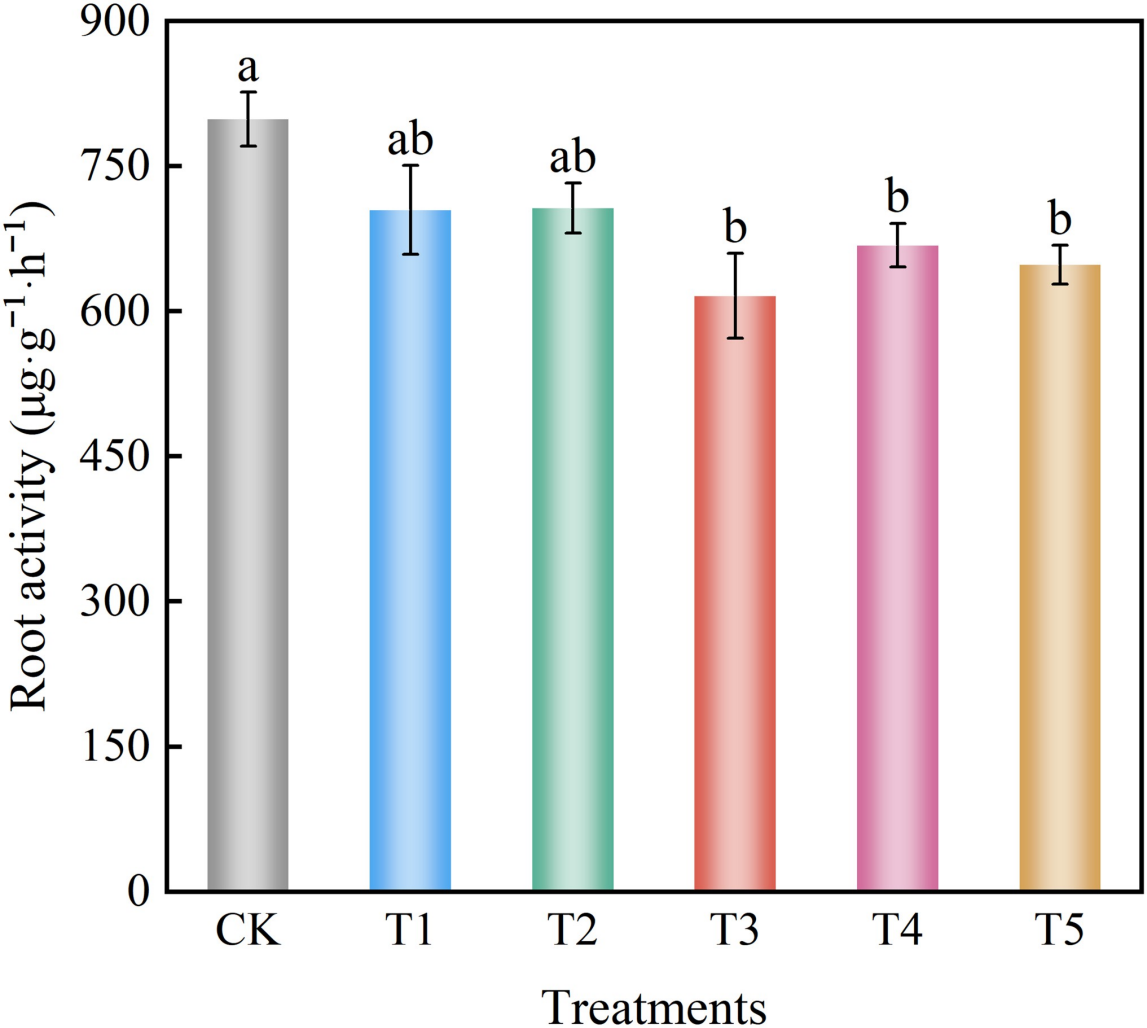
Effect of different nitrate levels on the root activity of spinach. Mean values with different alphabets differ significantly at (*p* < 0.05) by Duncan’s test.

The application of different nitrate levels decreased spinach root vigor (Fig 3). The root vigor of treatments T1 and T2 were not different from that of the control group. Treatments T3, T4, and T5 were significantly decreased compared to the control group by 22.88%, 16.31%, and 18.83%, respectively.

### Chloroplast pigment and photosynthetic system

#### Chloroplast pigment

**Fig 4:**
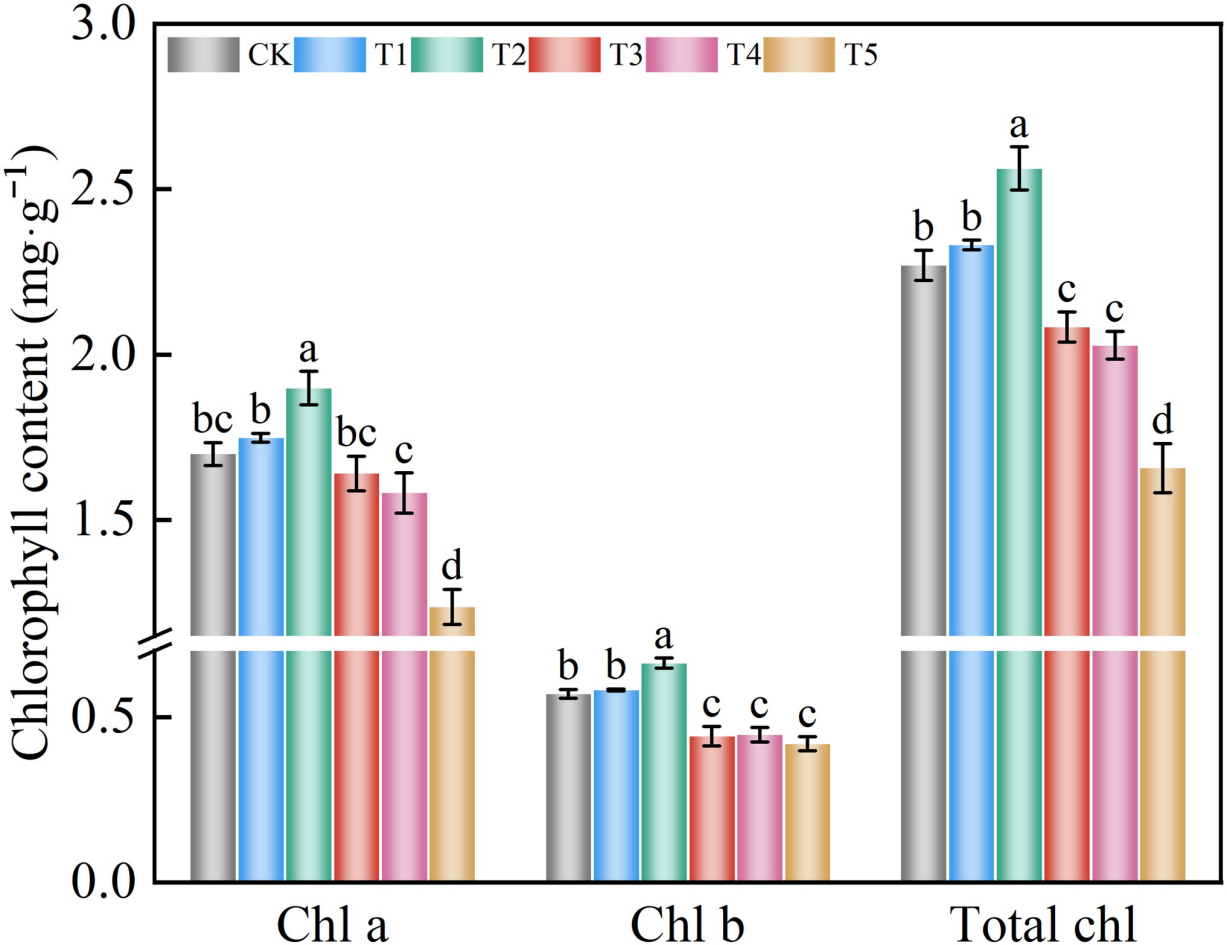
Effect of different nitrate levels on chlorophyll a, chlorophyll b, and total chlorophyll contents of spinach. Mean values with different alphabets differ significantly at (*p* < 0.05) by Duncan’s test.

The chlorophyll a, chlorophyll b, and total chlorophyll content in spinach first increased and then decreased with increasing nitrate concentration (Fig 4). Compared to the control group, the chlorophyll a, chlorophyll b, and total chlorophyll contents of treatment T2 significantly increased by 11.73%, 16.25%, and 12.87%, respectively. Treatment T5 significantly decreased the chlorophyll a content by 27.16% compared to the control group. The chlorophyll b content in treatments T3, T4, and T5 significantly decreased by 22.48%, 21.79%, and 26.53%, respectively, compared to the control group. The total chlorophyll content in treatments T3, T4, and T5 decreased significantly by 8.24%, 10.66%, and 27.01%, respectively, compared to the control group. These results suggest that the nitrate content could be a significant influencer of photosynthetic pigments in spinach.

### Gas exchange parameters

**Fig 5:**
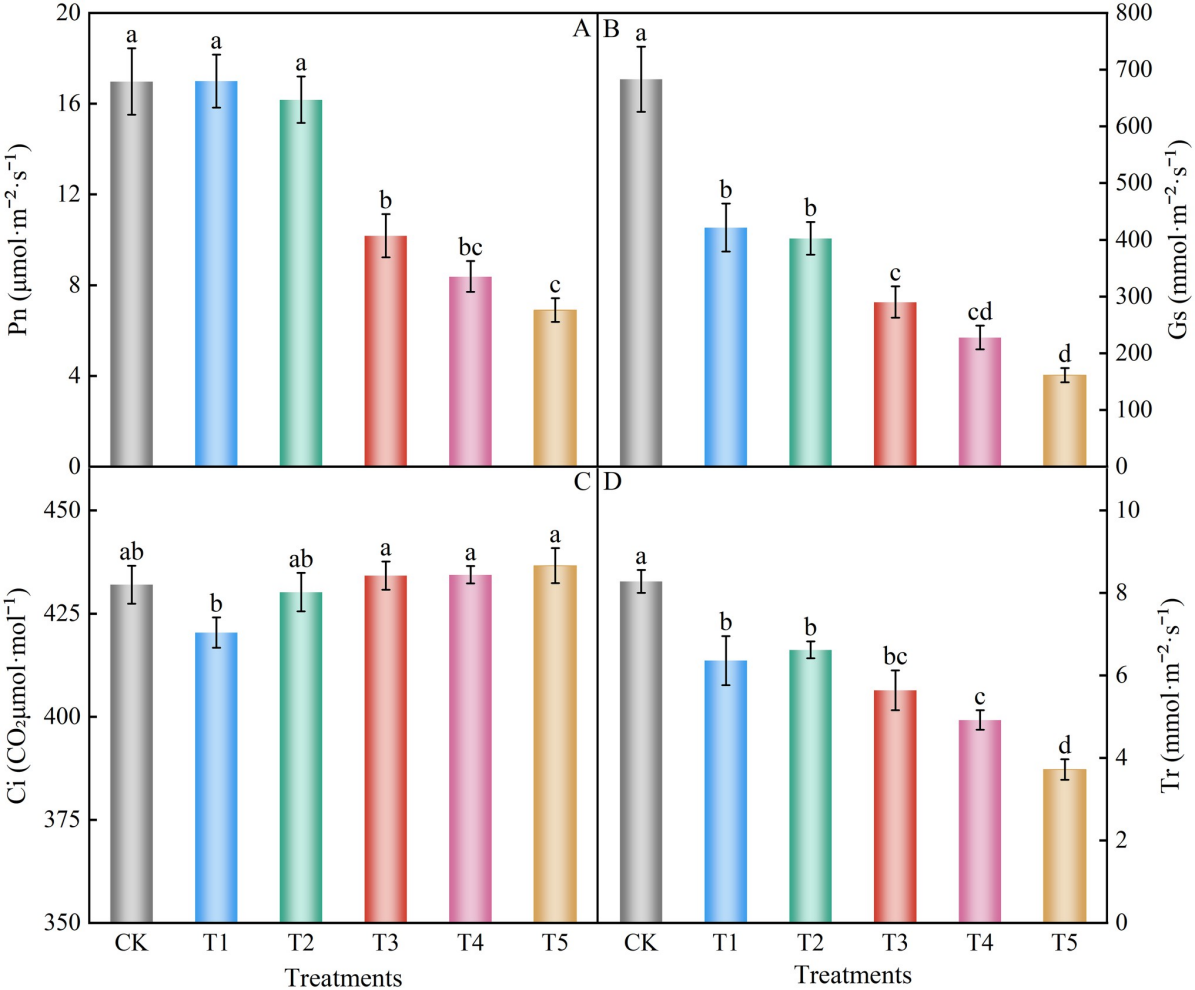
Effect of different nitrate levels on photosynthetic gas exchange parameters of spinach. Mean values with different alphabets differ significantly at (*p* < 0.05) by Duncan’s test. (A) Net photosynthetic rate, Pn. (B) Stomatal conductance, Gs. (C) Intercellular CO_2_ concentration, Ci. (D) Transpiration rate, Tr.

With increasing nitrate content, Pn, Gs, and Tr decreased to varying degrees. In contrast, the change in Ci was not significant (Fig 5). The Pn values (Fig 5A) of treatments T1 and T2 were not different from that of the control group. The Pn value decreased significantly when the nitrate level increased to 150 mM (T3 treatment). The Pn of treatments T3, T4, and T5 decreased significantly compared to the control group by 40.05%, 50.65%, and 59.36%, respectively. The Gs (Fig 5B) of treatments T1, T2, T3, T4, and T5 decreased significantly compared to control group by 38.29%, 41.07%, 57.52%, 66.66%, and 76.38%, respectively. The Ci (Fig 5C) showed a small decrease in the T1 treatment, but the difference was not significant compared to the control group, and then a small increase with increasing nitrate, but none of the differences were significant compared to the control group. Overall, there was no significant change in Ci with increasing nitrate concentrations. Tr (Fig 5D) of treatments T1, T2, T3, T4, and T5 decreased significantly compared to control group by 23.19%, 20.05%, 31.88%, 40.58%, and 55.07%, respectively. In conclusion, nitrate treatment decreased Pn, stomatal conductance, and transpiration rate of spinach without significant effects on intercellular CO_2_ concentration.

### Chlorophyll fluorescence parameters

**Fig 6:**
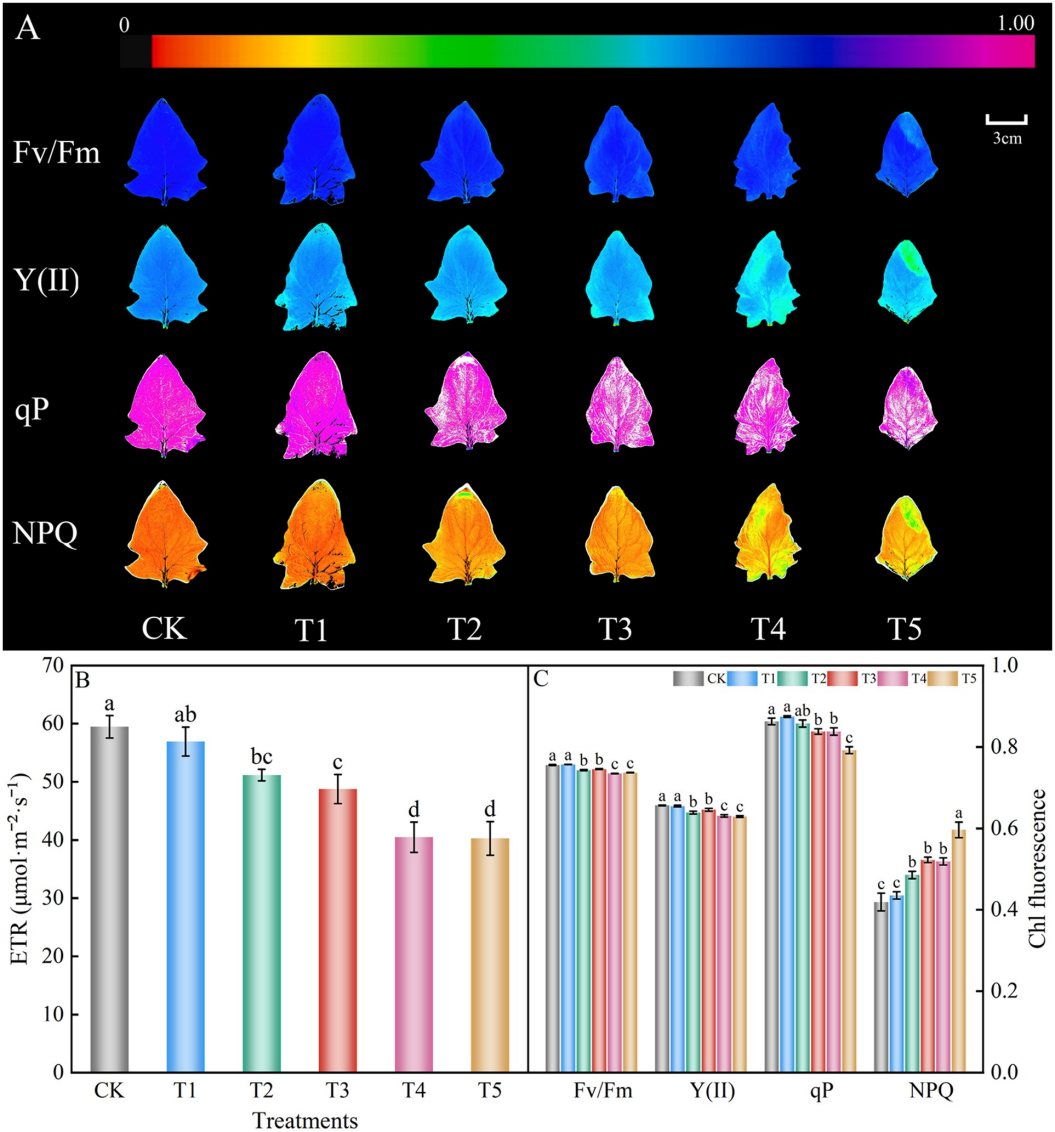
Effect of different nitrate levels on chlorophyll fluorescence parameters of spinach. Mean values with different alphabets differ significantly at (*p* < 0.05) by Duncan’s test. The coloured bar at the top of the leaf images (A) represents the chlorophyll fluorescence parameters range black (0) to purple (1.0) and how they mapped to the colour palette. (A) Images of Fv/Fm, Y(II), qP and NPQ, respectively. False colors are used to represent values of the parameter ranging from 0 (black) to 1.00 (purple). (B) The electron transport rates, ETR. (C) The maximum photochemical efficiency of PSΠ, Fv/Fm; PSII actual photochemical efficiency, Y(II); Photochemical quenching, qP; Non-photochemical quenching, NPQ.

The application of different amounts of nitrate decreased Fv/Fm, Y(II), qP, and ETR, and increased NPQ (Fig 6). The Fv/Fm, Y(II), qP, NPQ, and ETR of the T1 treatment were not significantly different from those of control group. Fv/Fm, Y(II), qP, and ETR of T3 treatment decreased significantly compared to the control group by 1.32%, 1.57%, 3.01%, and 17.99%, respectively, whereas NPQ increased by 24.70%. Fv/Fm, Y(II), qP, and ETR in the T5 treatment decreased significantly compared to the control group by 2.47%, 4.16%, 8.19%, and 32.29%, respectively, while NPQ increased significantly by 42.31%. These results suggest that the nitrate content can influence the photosynthetic performance of spinach.

### Nitrate content and NR activity

**Fig 7:**
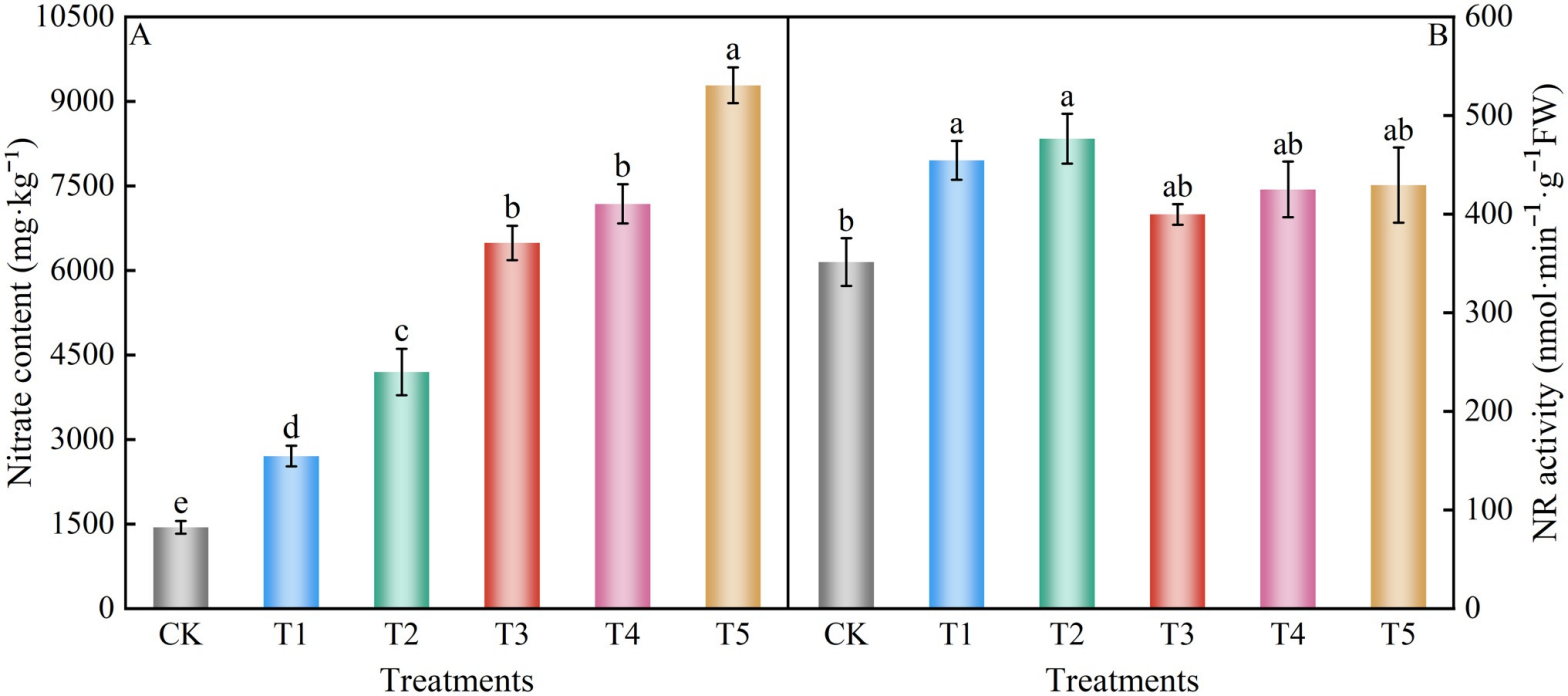
Effect of different nitrate levels on the accumulation of nitrate and NR activity of spinach leaves. Mean values with different alphabets differ significantly at (*p* < 0.05) by Duncan’s test.

NR is the key enzyme for converting nitrate-nitrogen to ammonium-nitrogen in plants. When plants ingest nitrate, it must be reduced metabolically by NR before being used by plants. Therefore, NR plays a key role in plant nitrogen metabolism. The accumulation of nitrate in spinach leaves increased with increasing nitrate content (Fig 7A). The accumulation of nitrate in the control group was the lowest at 1445.18 mg kg^-1^, which was within the range of the national standards for tolerance limits for nitrate in vegetables. The accumulation of nitrate in treatments T1, T2, T3, T4, and T5 increased significantly compared to control group (87.33%, 190.44%, 348.94%, 396.89%, and 542.49%, respectively). However, the differences between the T3 and T4 treatments were not significant. The activity of NR in spinach leaves showed a trend that first increased and then decreased with increasing nitrate levels (Fig 7B). All treatments were higher than in the control group, with increases of 29.41%, 35.63%, 13.79%, 20.99%, and 22.21%, respectively. However, T3, T4, and T5 treatments were not significantly different from the control. Thus, the presence of a certain amount of NO_3_^-^ induces NR activity.

### Contents of soluble protein, flavonoids, proline, and malondialdehyde

The soluble protein content was significantly higher in treatments T2 and T3 than in the control group by 2.34% and 2.59%, respectively. The application of nitrate at different concentrations initially increased the flavonoid content in spinach compared with that of the control group. The flavonoid content in treatments T2 and T3 significantly increased by 16.25% and 54.09%, respectively, compared to that of the control group. The flavonoid content in treatments T4 and T5 decreased significantly by 28.10% and 30.76%, respectively, compared to the control group. The proline content in spinach leaves and roots increased with increasing nitrate levels. The proline content in spinach leaves in treatments T2, T3, T4, and T5 significantly increased by 188.11%, 202.76%, 487.91%, and 956.63%, respectively, compared to the control group. The proline content in spinach roots in treatments T3, T4, and T5 significantly increased by 154.12%, 332.21%, and 560.14%, respectively, compared to the control group. It was measured that spinach, under nitrate stress, accumulates a high amount of proline, which is a highly desirable osmoregulatory substance that protects spinach growth under osmotic stress. The malondialdehyde content reflects the degree of damage to plants caused by salt stress. The malondialdehyde content in spinach leaves and roots showed an increasing trend with increasing nitrate levels. The malondialdehyde content in spinach leaves in treatments T2, T3, T4, and T5 significantly increased by 33.35%, 39.81%, 55.69%, and 43.76%, respectively, compared to the control group. The malondialdehyde content in spinach roots in treatments T2, T3, T4, and T5 significantly increased by 54.80%, 154.20%, 221.48%, and 269.00%, respectively, compared to the control group. It can be seen that the higher the nitrate concentration, the more the spinach was damaged (S4 Table).

### Antioxidant enzyme activity

**Fig 8:**
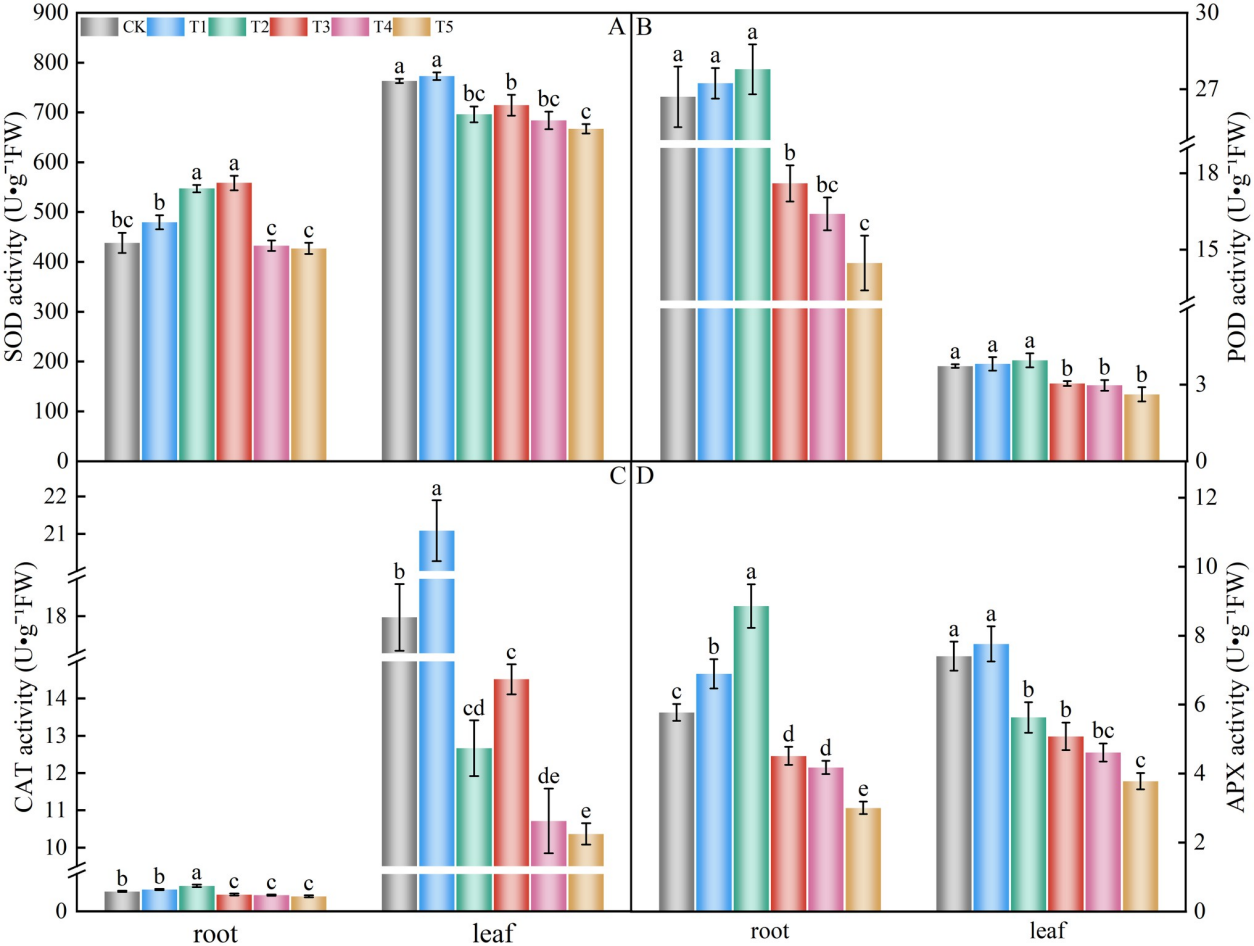
Effect of different nitrate levels on SOD, POD, CAT, and APX activities of spinach leaves and roots. Mean values with different alphabets differ significantly at (*p* < 0.05) by Duncan’s test. (A) superoxide dismutase, SOD. (B) peroxidase, POD. (C) catalase, CAT. (D) ascorbate peroxidase (APX).

SOD, POD, CAT, and APX are the major antioxidant enzymes that scavenge ROS and play essential roles in the antioxidant system of plants. SOD, POD, CAT, and APX activities of spinach leaves and roots treated with different amounts of nitrate showed a similar trend (Fig 8). The SOD activity of spinach roots (Fig 8A) of treatments T2 and T3 significantly increased compared to the control group by 24.87% and 27.47%, respectively. In contrast, the activity of leaves (Fig 8A) in treatments T2, T3, T4, and T5 significantly decreased compared to the control group (8.78%, 6.39%, 10.39%, and 12.60%, respectively). The POD activity of spinach roots and leaves (Fig 8B) in treatments T3, T4, and T5 were significantly lower than that of the control group by 34.07%, 38.54%, 45.79%, 18.39%, 20.27%, and 29.82%, respectively. Treatments T1 and T2 were higher than in the control group but did not differ significantly. The CAT activity of spinach roots (Fig 8C) was significantly higher than that of the control group in the T2 treatment, with an increase of 28.13%, and significantly lower than that of the control group in the T3, T4, and T5 treatments, with decreases of 15.63%, 18.75%, and 25.00%, respectively. The CAT activity of the leaves (Fig 8C) was significantly higher than that of the control group at the T1 treatment, with an increase of 17.35%, and significantly lower than that of the control group at T2, T3, T4, and T5 treatments, with decreases of 29.50%, 19.20%, 40.35%, and 42.30%, respectively. The APX activity of spinach roots (Fig 8D) was significantly higher than that of the control group in T1 and T2 treatments, with an increase of 19.40% and 53.46%, and significantly lower than in the control group in T3, T4, and T5 treatments, with a decrease of 21.82%, 27.60%, and 47.92%, respectively. The APX activity of leaves (Fig 8D) was significantly lower than in the control group in T2, T3, T4, and T5 treatments, with decreases of 24.03%, 31.41%, 37.71%, and 48.97%, respectively.

## Discussion

### Growth parameters

Salt is one of the most critical abiotic stressors that inhibit plant growth and development [33], and external morphology and growth status are the most visible indicators of the extent of salt damage to plants [34]. Salt stress has been shown to significantly inhibit the growth of the leaf length, leaf width, and leaf area of apple seedlings [35] and tropical lawns [36]. Chen et al. [37] showed that high nitrate stress reduced the leaf length, leaf width, leaf area, and biomass of alfalfa. The different nitrate levels in this experiment showed significant inhibition of spinach growth and reduced leaf length, width, and area compared to the control. Salt stress has a significant inhibitory effect on the biomass of wheat seedlings [38] and tomatoes [39]. Gou et al. [40] showed that nitrate stress significantly reduced the fresh and dry weights of cucumber seedlings. This experiment showed that nitrate stress inhibited the aboveground and belowground fresh weight and aboveground dry weight of spinach, which was generally consistent with the previous studies mentioned above.

Lv et al. [41] showed that low-N stress significantly increased the total root length, total surface area, and root tip number of wheat. In tobacco plants, low nitrate levels promote root growth, while the opposite promotes root growth [42]. Khan et al. [43] showed that the root growth of *Atriplex griffithii* var. *stocksii* showed substantial promotion at 90 mM NaCl; Bernstein & Kafkafi [44] showed that salinity limits the growth of the root system. This experiment showed that low nitrate levels had a promoting effect on total root length, total root volume, total root surface area, number of root tips, and number of branches of spinach. In contrast, high nitrate levels had an inhibitory effect, consistent with the results of previous studies mentioned above. Calcium nitrate stress has also been shown to inhibit the growth of pakchoi seedlings significantly [45], resulting in significant decreases in plant height, root length, leaf area, root volume, and plant dry and fresh weights. In summary, nitrate stress affects spinach growth and biomass accumulation, with low nitrate levels promoting root growth and above a specific range producing an inhibitory effect.

Root growth and vigor directly affect individual plant growth, nutritional status, and yield. Therefore, the root vigor index is an important indicator [46]. It has been shown that salt stress can significantly affect the root vigor of lettuce [47] and mulberry-grafted seedlings [48]. In this study, the root vigor of spinach decreased significantly when the NO_3_^-^ concentration reached 150 mM, and the difference was significant compared to the control. However, when treated with a low NO_3_^-^ concentration, the root vigor was not significantly different from that of the control. This may be because plants maintain root vigor at a specific range of NO_3_^-^ concentrations to adapt to external stress through their regulatory system. When the stress level exceeds the regulatory capacity of plants, root cell growth is delayed, and root vigor is reduced, which in turn impairs the uptake of water and mineral nutrients by plants, thus affecting normal plant growth [49].

### Chloroplast pigment and photosynthetic system

The chlorophyll content is an essential indicator of photosynthetic performance [50]. When plants are subjected to salt stress, various physiological processes directly or indirectly influence chlorophyll content and photosynthesis [51]. Gautam & Singh [52] found that the photosynthetic pigments in the leaves of maize seedlings decreased sharply with increasing salt stress. Akladious & Mohamed [53] reported that salt stress causes a significant reduction in chlorophyll a, chlorophyll b, carotenoids, and total chlorophyll in pepper plants. Previous studies have found that mild salt stress increases spinach chlorophyll content [54], whereas high salt (172 or 200 mM NaCl) reduces spinach chlorophyll content [55–57]. This experiment also showed that low nitrate levels increased the chlorophyll content in spinach, whereas high nitrate levels resulted in a significant decrease in chlorophyll content. This could be because the integrity of the chloroplast vesicle membrane is damaged under nitrate stress, which accelerates the degradation of photosynthetic pigments, weakening the photosynthetic efficiency [58].

Photosynthesis is crucial in plant growth and development and is the basis for plant survival. It also plays an essential mediating role in the carbon and oxygen cycles of Earth [59]. The mechanism of damage to plant photosynthesis under nitrate stress is not fully understood. Under saline conditions, salts accumulate in large amounts in the soil and cause osmotic stress and disruption of nutrient ion homeostasis in plants, which in turn affects the metabolic function of plant cells, reduces the photosynthetic capacity of plants, and inhibits plant growth, development, and productivity [59]. When plants are exposed to salt stress, leaf stomata contract, and Gs decrease, limiting the transport of CO_2_ to chloroplasts and the evaporation of leaf water, which in turn inhibits leaf photosynthesis and transpiration [60]. Plant photosynthetic capacity is limited by both stomatal and non-stomatal factors [61]. When Gs and Ci decrease, the weakening of Pn may be caused by the limitation of the stomatal factors. When Gs decreases and Ci remains largely unchanged, or even increases, the weakening of Pn may be caused by the limitation of non-stomatal factors, including the external environment, affecting the assimilative capacity of leaf pith cells, or the deterioration of photosynthetic performance [62, 63]. In this study, nitrate stress decreased Pn and Gs, while Ci slightly increased. However, the difference was not significant compared with the control group, indicating that nitrate stress may reduce the photosynthetic capacity of spinach by limiting non-stomatal factors [63]. Lan et al. [64] showed that a reduction in the photosynthetic rate of cucumber under nitrate stress was due to non-stomatal limitation, which negatively affected plant growth, consistent with the results of the present study. The results of this study showed that a turning point Pn occurred when the NO_3_^-^ concentration was 150 mM. Pn decreased significantly when the NO_3_^-^ concentration further increased. One reason for this change could be the decrease in chlorophyll content and osmotic stress caused by the high NO_3_^-^ concentration, which led to a decrease in the photosynthetic rate [65]. It was also shown that the photosynthetic gas exchange parameters of pakchoi [66] and *Isatis indigotica* Fort [67] were significantly reduced, and plant growth was inhibited under salt stress. It was also shown that salt stress significantly reduced Pn, Gs, and Tr and increased Ci in the leaves of lettuce seedlings, indicating that the decrease in photosynthesis was mainly due to non-stomatal factors, implying that the decrease in photosynthetic activity of leaf pith cells caused a decrease in photosynthesis [51]. This was consistent with the results of this experiment.

Plant chlorophyll fluorescence is closely related to photosynthetic efficiency and may reflect the degree of damage to plant photosynthesis by stress, and it is also known as the internal probe of photosynthesis [68]. Among the chlorophyll fluorescence parameters, Fv/Fm indicates the maximal quantum yield of PSII photochemistry, which is an efficient indicator of the degree of PSII photoinhibition [62]. Y(II) is the quantum efficiency of PSII photochemistry, which can be used to characterize the photosynthetic capacity of plants [69]. The ETR is a parameter that indicates the magnitude of the photosynthetic capacity of plants and reflects the apparent electron transfer rate of plants under actual light intensity [70]. QP is the photochemical quenching coefficient, which reflects the share of light energy absorbed by PSII antenna pigments for photochemical electron transfer, and represents the degree of opening of the PSII reaction center [71]. The non-photochemical quenching coefficient NPQ indicates that the light energy absorbed by the antenna pigment cannot be used for photochemical electron transfer but for thermal dissipation [72]. The larger the NPQ, the smaller the damage to the PSII reaction center [62]. Important information about PSII can be obtained by analyzing chlorophyll fluorescence parameters [73]. Khaled Al-Taweel & Wadano [74] showed that salt stress impairs PSII repair, thereby enhancing photoinhibition. In addition, Athar et al. [75] reported that salt stress reduces the efficiency of transferring energy absorbed by antenna chlorophyll a to the reaction center of PSII and damages or dissociates light-harvesting proteins. Lan et al. [64] showed that the levels of Fv/Fm, Fv’/Fm’, qP, and ETR were reduced and the levels of qN increased in cucumber leaves under nitrate stress, indicating that stress inhibits the uptake and utilization of light energy and the rate of electron transfer and increases the heat release of light energy. Spinach plants prevent light damage by dissipating heat, which can be learned using NPQ [73]. In this experiment, Fv/Fm, Y(II), qP, and ETR of spinach leaves gradually decreased, and NPQ gradually increased when the nitrate concentration gradually increased. This experiment showed that the application of nitrate stress weakened the uptake of light energy and the conversion efficiency of spinach leaves, inhibited the electron transfer of PSII reaction centers, and weakened photosynthesis, thus inhibiting plant growth and development. This is in general agreement with the trends observed in photosynthesis.

### Nitrate content and NR activity

The nitrate content is an efficient index for evaluating the quality of vegetables [76]. Numerous studies have shown that the amount of nitrogen fertilizer promotes the accumulation of nitrate in vegetables to some extent and that nitrogen content is positively correlated with nitrate content [77]. This experiment showed that nitrate stress significantly increased the nitrate content in spinach, which is consistent with the results of previous studies. NR is a crucial enzyme for the conversion of nitrate to ammonium nitrogen in plants [78], which has a relevant effect on plant growth and development, yield, and quality. In this experiment, the activity of NR in spinach leaves first increased and then decreased with increasing nitrate levels, but in all cases, it was higher than that of the control. This is consistent with findings on salt stress in *Thellungiella halophila* [79], where nitrate reductase activity increased and then decreased with increasing salt concentration.

### Osmotic adjustment and antioxidation

Soluble proteins are important osmoregulators in plants [80]. Numerous studies have shown that soluble protein content is significantly increased when plants are subjected to osmotic stress to maintain the osmotic balance of plant cells, thus mitigating damage to the plant [81]. In this study, osmotic stress due to nitrate increased soluble protein content. Flavonoids in vegetables scavenge free radicals and increase the antioxidant capacity of plants [82]. Studies have shown that the flavonoid content in plants is affected by nitrogen content [83]. In this experiment, low nitrate levels increased flavonoid content in spinach, whereas high levels decreased flavonoid content. This may be because spinach accumulates flavonoids within a specific range through its own regulatory ability to scavenge free radicals and improve its antioxidant capacity. At the same time, high levels of nitrate can disrupt this regulation. Studies have shown that proline is a crucial osmoregulatory compound in plants. When plants are exposed to osmotic stress, proline accumulates in large amounts to maintain water balance, regulate intracellular osmotic pressure, and stabilize the structure of biomolecules [84, 85]. This experiment showed that the proline content in spinach roots and leaves increased significantly with increasing treatment concentrations, indicating that cellular water uptake became more complex, and plants balanced osmotic pressure by accumulating a large amount of proline. This is consistent with the results of previous studies on cucumber [86] and tomato seedlings [87] under nitrate stress. Studies have shown that salt stress leads to a considerable accumulation of reactive oxygen species in plants and causes peroxidative damage to cell membrane lipids, resulting in higher MDA content in plants, the level of which may reflect, to some extent, the extent of cell membrane damage [88, 89]. It was found that nitrate stress increased malondialdehyde content and membrane lipid peroxidation in cucumber roots and leaves [86, 90]. In this study, nitrate stress significantly increased the MDA content in spinach roots and leaves, suggesting that nitrate stress disrupts the integrity of the cell membrane system and increases the extent of membrane lipid peroxidation.

Antioxidant enzyme protection systems act synergistically in plants to protect cell membranes from reactive oxygen species, inhibit membrane lipid peroxidation, and allow normal plant growth and metabolism [91]. Treatment with low NaCl concentration increased the activity of antioxidant enzymes and promoted the growth of *Thellungiella halophila*, whereas treatment with high NaCl concentration caused a decrease in SOD activity [92]. In this experiment, low nitrate levels caused higher SOD, POD, CAT, and APX activities in spinach roots and leaves than in the control group, which might be one of the stress responses of plants to resist the stress. High nitrate levels significantly decreased the activity of antioxidant enzymes in the roots and leaves of spinach. This is consistent with the results in tomatoes under salinity stress, where low salinity concentrations increased SOD and CAT activities, while high concentrations decreased enzyme activities [93]. However, there were differences in the changes in SOD, POD, CAT, and APX activities, which all decreased in tomato seeds [94] and cucumber [86] under nitrate stress. This experiment showed that SOD, POD, CAT, and APX are coordinated under stress conditions to maintain low oxygen species in plants and ensure normal plant growth; however, there is a limit to the effect of the antioxidant enzyme protection system. At low concentrations, antioxidant enzymes in plants can independently scavenge reactive oxygen, and enzyme activity increases. Once this range is exceeded, the enzyme protection system is damaged and activity decreases. Therefore, the reactive oxygen species can no longer be scavenged, and the antioxidant capacity of plants weakens.

## Conclusions

This study investigated the effects of different nitrate levels on the growth, physiology, and photosynthesis of spinach. The results showed that applying nitrate decreased root vigor, photosynthesis, and inhibited the photochemical efficiency of PSII. However, it increased the NR activity of leaves and the concentration of osmoregulatory substances, showing some nitrate tolerance. Nonetheless, low levels of nitrate promoted spinach morphology and root growth. Our study shows that plant responses to nitrate are concentration-dependent, suggesting that plant-specific concentrations of salts might be beneficial. Therefore, plant-specific nitrate tolerance can promote desirable agronomic traits, even if they are determined by various physiological and metabolic pathways.

## Supporting information

S1 Table Effect of different nitrate levels on leaf length, leaf width, and leaf area of spinach. (DOCX)

S2 Table Effect of different nitrate levels on the biomass of spinach. (DOCX)

S3 Table Effect of different nitrate levels on the morphological parameters of spinach roots. (DOCX)

S4 Table Effect of different nitrate levels on soluble protein, flavonoid, proline and MDA contents of spinach. (DOCX)
