# Supplementary material for: Changes in growth, physiology, and photosynthetic capacity of spinach (Spinacia oleracea L.) under different nitrate levels
Source: PLoS One. 2023 Mar 31;18(3):e0283787. doi: 10.1371/journal.pone.0283787 (PMC10065267; doi:10.1371/journal.pone.0283787)
Supplement: S1 Table — (DOCX) [file pone.0283787.s001.docx]

| Treatment | Leaf length (cm) | Leaf width (cm) | Leaf area (cm²) |
| --- | --- | --- | --- |
| CK | 7.78±0.10a | 5.30±0.07ab | 78.63±4.07a |
| T1 | 7.28±0.07b | 5.36±0.11a | 78.04±6.58a |
| T2 | 7.06±0.21bc | 4.90±0.13bc | 69.77±2.22ab |
| T3 | 6.98±0.18bc | 4.86±0.18c | 69.57±5.78ab |
| T4 | 6.74±0.20cd | 4.78±0.19c | 59.12±5.23bc |
| T5 | 6.36±0.15d | 3.80±0.14d | 51.84±1.89c |
