# Supplementary material for: Changes in growth, physiology, and photosynthetic capacity of spinach (Spinacia oleracea L.) under different nitrate levels
Source: PLoS One. 2023 Mar 31;18(3):e0283787. doi: 10.1371/journal.pone.0283787 (PMC10065267; doi:10.1371/journal.pone.0283787)
Supplement: S2 Table — (DOCX) [file pone.0283787.s002.docx]

| Treatment | Fresh weight (g) | | Dry weight (g) | | Relative moisture content (%) |
| --- | --- | --- | --- | --- | --- |
|  | Shoot | Root | Shoot | Root |  |
| CK | 12.50±0.95a | 3.70±0.21ab | 1.26±0.08a | 0.29±0.02a | 90.41±0.23a |
| T1 | 10.87±0.64ab | 4.03±0.15a | 1.22±0.10ab | 0.28±0.02a | 89.95±0.32ab |
| T2 | 9.27±0.56bc | 3.97±0.12a | 1.19±0.08ab | 0.32±0.03a | 88.63±0.20bc |
| T3 | 7.90±0.20c | 3.53±0.19ab | 1.07±0.08ab | 0.31±0.02a | 87.94±0.51c |
| T4 | 7.93±0.68c | 3.30±0.12bc | 1.06±0.02ab | 0.32±0.02a | 87.54±0.74c |
| T5 | 7.80±0.42c | 2.97±0.12c | 1.01±0.03b | 0.33±0.02a | 87.53±0.73c |
