# Supplementary material for: Changes in growth, physiology, and photosynthetic capacity of spinach (Spinacia oleracea L.) under different nitrate levels
Source: PLoS One. 2023 Mar 31;18(3):e0283787. doi: 10.1371/journal.pone.0283787 (PMC10065267; doi:10.1371/journal.pone.0283787)
Supplement: S3 Table — (DOCX) [file pone.0283787.s003.docx]

| Treatment | Total root  length (cm) | Total root volume (cm^3^) | Total root surface area (cm²) | The number of root tips | Branch number |
| --- | --- | --- | --- | --- | --- |
| CK | 527.34±18.91b | 2.96±0.09b | 141.93±6.86b | 317.67±7.84b | 3510.00±201.28b |
| T1 | 669.86±47.62a | 3.70±0.01a | 175.90±10.21a | 284.67±13.38bc | 4364.33±282.82a |
| T2 | 678.07±42.86a | 3.47±0.10a | 171.93±5.70a | 357.33±14.11a | 4545.67±288.67a |
| T3 | 430.05±15.02c | 2.67±0.10c | 125.35±4.20bc | 287.00±11.59bc | 3348.67±70.94b |
| T4 | 347.77±29.54cd | 2.55±0.12cd | 105.22±8.45cd | 257.33±12.41c | 2391.00±131.15c |
| T5 | 274.38±20.41d | 2.37±0.05d | 90.11±3.46d | 205.67±11.35d | 1788.33±71.26c |
