# Supplementary material for: Changes in growth, physiology, and photosynthetic capacity of spinach (Spinacia oleracea L.) under different nitrate levels
Source: PLoS One. 2023 Mar 31;18(3):e0283787. doi: 10.1371/journal.pone.0283787 (PMC10065267; doi:10.1371/journal.pone.0283787)
Supplement: S4 Table — (DOCX) [file pone.0283787.s004.docx]

| Treatment | Soluble protein  (mg·g^-1^ FW) | Flavonoid  (μg·g^-1^ DW) | Proline (ug·g^-1^ FW) | | MDA (nmol·g^-1^ FW) | |
| --- | --- | --- | --- | --- | --- | --- |
|  |  |  | root | leaf | root | leaf |
| CK | 2.88±0.03bc | 10.89±0.24c | 18.44±0.58d | 50.36±0.34d | 1.06±0.07e | 6.30±0.24d |
| T1 | 2.86±0.02c | 10.07±0.70c | 24.33±1.19d | 75.38±2.22d | 1.32±0.09e | 7.40±0.13cd |
| T2 | 2.95±0.02a | 12.66±0.26b | 24.46±0.76d | 145.09±1.57c | 1.64±0.02d | 8.40±0.50bc |
| T3 | 2.95±0.02a | 16.78±0.25a | 46.86±2.91c | 152.47±4.08c | 2.69±0.02c | 8.80±0.57ab |
| T4 | 2.94±0.03ab | 7.83±0.40d | 79.70±5.32b | 296.08±23.44b | 3.40±0.17b | 9.80±0.16a |
| T5 | 2.88±0.01bc | 7.54±0.46d | 121.73±11.18a | 532.12±13.01a | 3.89±0.11a | 9.05±0.44ab |
